# Sea Turtles in the Cancer Risk Landscape: A Global Meta-Analysis of Fibropapillomatosis Prevalence and Associated Risk Factors

**DOI:** 10.3390/pathogens10101295

**Published:** 2021-10-08

**Authors:** Antoine M. Dujon, Gail Schofield, Roberto M. Venegas, Frédéric Thomas, Beata Ujvari

**Affiliations:** 1 Centre for Integrative Ecology, School of Life and Environmental Sciences, Deakin University, Geelong Waurn Ponds, Vic 3216, Australia; rvenegas@deakin.edu.au (R.M.V.); beata.ujvari@deakin.edu.au (B.U.); 2 CANECEV-Centre de Recherches Ecologiques et Evolutives sur le Cancer (CREEC), 34090 Montpellier, France; frederic.thomas2@ird.fr; 3 MIVEGEC, CREEC, UMR IRD 224-CNRS 5290-Université de Montpellier, 34295 Montpellier, France; 4 School of Biological and Chemical Sciences, Queen Mary University of London, London E1 4NS, UK; g.schof@gmail.com

**Keywords:** epidemiology, neoplasm, landscape ecology, conservation, cumulative impact, climate change, pollution

## Abstract

Several cancer risk factors (exposure to ultraviolet-B, pollution, toxins and pathogens) have been identified for wildlife, to form a “cancer risk landscape.” However, information remains limited on how the spatiotemporal variability of these factors impacts the prevalence of cancer in wildlife. Here, we evaluated the cancer risk landscape at 49 foraging sites of the globally distributed green turtle (*Chelonia mydas*), a species affected by fibropapillomatosis, by integrating data from a global meta-analysis of 31 publications (1994–2019). Evaluated risk factors included ultraviolet light exposure, eutrophication, toxic phytoplanktonic blooms, sea surface temperature, and the presence of mechanical vectors (parasites and symbiotic species). Prevalence was highest in areas where nutrient concentrations facilitated the emergence of toxic phytoplankton blooms. In contrast, ultraviolet light exposure and the presence of parasitic and/or symbiotic species did not appear to impact disease prevalence. Our results indicate that, to counter outbreaks of fibropapillomatosis, management actions that reduce eutrophication in foraging areas should be implemented.

## 1. Introduction

Cancers are a group of diseases that emerge in multicellular organism hosts when cells stop collaborating with surrounding cells, and obtain the ability to proliferate in an uncontrolled manner [1,2]. The emergence of cancers mirrors speciation events, in which new parasitic species emerge and consume the resources of hosts, reducing overall fitness [3,4,5]. Cancers have the potential to drive already threatened wildlife towards extinction (i.e., the Tasmanian devil, *Sarcophilus harrisii*, [6]; California sea lion, *Zalophus californianus*, [7]; Santa Catalina fox, *Urocyon littoralis catalinae*, [8]; Cape mountain Zebra, *Equus zebra zebra*, [9]). Cancers can also cause significant changes to the ecosystems of impacted wildlife through trophic cascades and reshaping of biotic interactions, leading to mass mortalities [10,11,12,13,14,15]. For example, one transmissible cancer depleted > 80% of Tasmanian devils, a keystone predator, from the ecosystem of the island of Tasmania (Australia) [16]. This catastrophic event caused the complete reorganization of the trophic chain, releasing meso-predators from competition with Tasmanian devils, and allowing invasive species, such as feral cats (*Felis catus*), to proliferate [11,12]. 

Thus, it is important to quantify the impact of cancers on wildlife populations and associated ecosystems [17,18,19,20]. For instance, several studies have delineated cancer-causing risk factors for wildlife populations [19,20,21], which are directly associated with increasing anthropogenic pressures in both terrestrial and aquatic ecosystems [22]. These risk factors, which include exposure to chemicals, ultraviolet light, ionizing particles, and habitat fragmentation, form a landscape in which species are exposed to cumulative impacts of varying spatiotemporal variability [23]. Thus, evaluating historical data within this framework provides an opportunity to examine the drivers of cancer in wildlife.

Sea turtles are a group of seven threatened species at risk of various diseases, including a type of cancer called fibropapillomatosis [24,25,26]. This disease was first documented in Key West, Florida, USA in 1938 in green turtles (*Chelonia mydas*, [27]), but has now been detected in all seven sea turtle species and across all ocean basins in which the species are present, reaching panzoonotic status [25,26]. Fibropapillomatosis manifests as cutaneous tumors on the body surface and internal organs [24,28]. Fibropapillomatosis is thought to be initiated by the Chelonid Alphaherpesvirus 5 (ChHV5, also known as Chelonid herpesvirus 5 in the literature) [29,30,31], and is likely horizontally transmitted via shedding of virus particles contained in epithelial cells, and possibly in urine and/or bodily secretions [29,32].

Fibropapillomatosis tumors range in size from 0.1 cm to greater than 30 cm in diameter [24]. These tumors debilitate the host by impeding movement and foraging ability, and reducing host immune function, while consuming energetic resources [33,34]. These tumors can be concomitant to other parasitic infections (i.e., trematodes [28]), but also become secondary infection sites for other parasitic species (i.e., bacterial infections, [35]). All of these factors increase the risk of death to individuals with this cancer [24,26,36]. However, not all sea turtles infected with the virus develop tumors, with regression and recovery also being possible [25,37,38]. Therefore, this disease is likely multifactorial [39], with sea turtles possibly being more likely to develop the disease when occupying stressful environments that exacerbate immunosuppression and inflammation [40], which are both tumor promotors. Because sea turtles contribute to ecosystem functioning, in addition to being of economic (e.g., wildlife watching) and cultural importance, it is important to quantify the risk of this disease to population resilience. Such knowledge could then be integrated in risk assessments to implement appropriate protection actions [41,42].

Here, we conducted a comprehensive meta-analysis on the prevalence of fibropapillomatosis in green turtles on a global scale. Out of the seven sea turtle species, green turtles have the overall highest prevalence of fibropapillomatosis, the largest number of publications on this subject, and some epidemiological studies spanning decades [28,43,44,45], making them the most suitable for meta-analysis. We used mixed-effect models to identify the risk factors most closely associated with the prevalence of fibropapillomatosis across populations. Our results are expected to demonstrate the importance of delineating the cancer risk landscape for integration in the conservation management of threatened species.

## 2. Material and Methods

### 2.1. Literature Review and Data Consolidation

Using a previously established protocol (see [46,47]), we performed a comprehensive literature review. We searched the Thomson Reuters ISI Web of ScienceTM database, the Scopus database, and Google Scholar for relevant publications spanning 1994–2019. Specific terms were used in the topic field, which included the title, abstract, keywords and keywords plus (i.e., words that frequently appear in the titles of articles cited within a publication). The terms included: “sea turtle”, “green turtle”, “*Chelonia mydas*”, “cancer”, “fibropapilloma”, “fibropapillomatosis”, and “neoplasia”. To locate potential articles missed by the initial search, we checked the reference and citation lists of relevant papers based on the predefined keywords. We also screened and included conference proceedings and reports in French, English, and Spanish [48]. See references [49,50,51,52,53,54,55,56,57,58,59,60,61,62,63,64,65,66,67,68,69,70,71,72,73,74,75,76] and Supplementary Table S1 for the full list of publications included in the meta-analysis.

For each document, which contained at least one year of data, we extracted the sample size (expressed as number of green turtles surveyed), the number of individuals infected with fibropapillomatosis, coordinates of the study sites (approximated using Google Earth if exact coordinates were not available in the publication), and the year(s) of sampling. We focused only on foraging sites, where sea turtles spend the majority of their immature and adult life-stages; thus, representing where they are most likely to be infected by the virus (see [77]). We also classified each study depending on the condition of the surveyed sea turtles, i.e., “stranded” (alive or dead on a beach), “live” (free in the water), or “mix” (if the survey contained a mixture of live, free, and dead or stranded turtles in the water or on the beach at the study sites). Measurements of sea turtle size (i.e., carapace length) were not consistently reported across studies (i.e., curved, straight, or estimated lengths), and a breakdown per year was rarely available; thus, we classified individuals into three size groups: “immature”, “adult”, or “mix” (if both groups were present) based on the information provided in each publication. Since size at maturity varies across sites and ocean basins, we relied on the classification provided by the authors and experts of each publication included in this meta-analysis. For many studies, it was not possible to distinguish between male and female turtles; therefore, they were grouped as adults only. There is currently no evidence in the literature that males and females are affected differently by ChHV5 [26]. A publication was excluded from the meta-analysis if it lacked any of the specified key information, and if the reported prevalence was computed by aggregating individuals sampled over a period of time of more than two adjacent years.

### 2.2. Identification of Potential Risk Factors

To define the cancer risk landscape of green turtles, we identified potential risk factors associated with fibropapillomatosis from the published literature [23]. We then obtained data for those risk factors from various online databases (where available) to investigate their effect on the prevalence of fibropapillomatosis in sea turtle foraging areas.

#### 2.2.1. Exposure to the Chelonid Herpesvirus 5 Virus

ChHV5 is strongly suspected to cause fibropapillomatosis in sea turtles, although Koch’s postulates have yet to be definitively confirmed because of difficulty in culturing the virus in a laboratory setting [78] (but see recent progress [79]). Laboratory experiments showed that green turtles develop tumors between 15 and 43 weeks after being exposed to cell-free tumor extracts [29]. Thus, sea turtles infected by ChHV5 can be observed in the wild that do not yet present tumors [25,38]. Since ChHV5 is globally distributed, we assumed that all populations included in this study had been exposed [25,30].

#### 2.2.2. Ultraviolet Light

Ultraviolet light (UV), especially UVB, is a well-documented cancer risk factor causing damage to DNA and increasing its mutation rate, including in aquatic ecosystems [80,81]. In at least one publication, UV was suspected to contribute to the emergence of tumors in ChHV5-infected sea turtles [82]; however, this hypothesis was not tested at a global scale. To quantify exposure to UV, annual minimum, maximum, and mean exposure data, along with seasonal data (all expressed in J·m^−2^·day^−1^), were obtained from the global UV-B radiation dataset for macroecological studies (glUV). This dataset included a series of climate surfaces containing information on various aspects of the global distribution of ultraviolet-B radiation [80].

#### 2.2.3. Seabed Depth

Green turtles are primarily benthic foragers feeding on algae and seagrass beds to depths typically up to 40 m [83,84]. Using the ETOPO1 1 Arc-Minute Global Relief Model (https://www.ngdc.noaa.gov/mgg/global accessed on 25 July 2021), the mean and median seabed depth of each study area was calculated, restricting the analysis to depths < 40 m (the area most likely frequented by turtles). Seabed depth is an interesting variable for inclusion here, because UV light is rapidly absorbed as sea water depth increases. Ninety-percent of the irradiance of UV is absorbed in the first 0.2 to 15 m of the water column, depending on the type of marine ecosystem [85].

#### 2.2.4. Eutrophication

Eutrophication, the excessive richness of nutrients [86,87] presents a potential risk factor for fibropapillomatosis because it promotes the growth of algae and phytoplankton that produce tumor-promoting components, such as biotoxins [88,89,90,91]. For example, exposure to biotoxins produced by benthic filamentous cyanobacterium (*Lyngbya majuscule*) and dinoflagellates (*Prorocentrum* spp.), as well as brevetoxins produced by red tides, represent potential risk factors for fibropapillomatosis [88,89,90,91].

To quantify eutrophication in the marine ecosystem, monthly surface concentrations of nitrate (NO^3−^), phosphorus (PO_4_^−3^), silicate (Si), and phytoplankton (in µmol·L^−1^) were obtained at a resolution of ¼° from the global ocean biogeochemistry hindcast (GLOBAL_REANALYSIS_BIO_001_029, https://resources.marine.copernicus.eu accessed on 25 July 2021) for the period between 1993 to 2019. Datasets on nitrate, phosphate, silicate, and phytoplankton concentrations were assimilated to compute metrics on an annual time scale, including minimum, maximum, mean, median, and standard deviations.

#### 2.2.5. Sea Surface Temperature

Transfection experiments in green turtles suggest that temperature influences the speed at which tumors appear after infection with ChHV5 [29]. Page-Kajan et al. [36] found that green turtles at rehabilitation centers developed more tumors during warmer months. In addition, a rise in temperature often increases the virulence of herpesviruses in other aquatic species (e.g., [92,93]). Temperature also impacts tumor growth in other poikilothermic species. For example, relatively cold temperatures (< 10 °C) promote the growth of papilloma tumors in the Japanese fire belly newt (*Cynops pyrrhogaster,* [94]), while relatively warm temperatures (> 10 °C) promote the growth of dermal sarcoma tumors in walleye (*Stizostedion vitreum,* [95]). Thus, temperature likely impacts fibropapillomatosis occurrence in sea turtles.

We obtained global daily sea surface temperature data (SST, expressed as degrees Celsius) from the Optimum Interpolation Sea Surface Temperature (OISST) v2.1 website (https://www.ncdc.noaa.gov/oisst/data-access accessed on 25 July 2021). The OISST dataset extends from September 1981 to the present at a resolution of ¼° [96]. The daily SST dataset was assimilated to compute the following metrics on an annual time scale: minimum, maximum, mean, median, and standard deviation. To determine a threshold for a potential effect of temperature, we also computed the number of days in each year with an SST greater than a given threshold, from 20 °C to 30 °C (using 1 °C steps).

#### 2.2.6. Potential ChHV5 Vectors

While horizontal transmission most likely occurs directly between individual green turtles through the shedding of viral particles [77], several species have been identified as potential ChHV5 mechanical vectors. At least two taxa represent potential mechanical vectors of ChHV5 that are able to infect sea turtles. Specifically, leeches of the genus *Ozobranchus* that attach to sea turtles are present in the Caribbean Sea and the Gulf of Mexico, while saddleback wrasse (*Thalassoma duperrey*) are present in the Hawaiian archipelago. Polymerase chain reaction (PCR) diagnostics confirmed that individuals of these two taxa carry high ChHV5 loads [97,98,99,100]. We obtained the global geographical distribution of both taxa from www.marinespecies.org accessed on 25 July 2021 and https://www.fishbase.se accessed on 25 July 2021. We classified the study sites as “present” and “absent” for these two taxa.

### 2.3. Data Consolidation and Spatial Scale Selection

Most published studies on green turtles were conducted at the spatial scale of a bay (up to ~80 km coastline, e.g., Moreton Bay in Australia [76]), island (up to ~85 km coastline, e.g., in La Martinique, France [66]) or along a relatively long stretch of coastline (up to ~60 km coastline, e.g., in Brazil [70]). Thus, to facilitate comparison, we computed the potential risk factors at a consistent scale, by calculating the metric of interest (min, max, mean, median, and standard deviation, etc.) of the values within two circles of ½° and 1° diameter around the coordinates of each study site, encompassing all study site scales. The environmental values associated to each prevalence measurement were computed for a time period starting one year before the start of a study to its end (over the 1994–2019 period). This approach took into account the fact that 15 to 45 weeks are required for tumors to develop in sea turtles observed with fibropapillomatosis after infection [29]. There was good agreement between the computed variables for the two spatial scales (92% of pairwise comparisons had a Pearson’s R > 0.85). Thus, we conducted all subsequent statistical analyses using the metrics computed at a 1° spatial scale. 

### 2.4. Statistical Analysis

We used a series of logistic mixed effect regression models to quantify the effects of the potential risk factors on the prevalence of fibropapillomatosis [101]. All models used in our study were mixed-effect models. To avoid losing temporal resolution, we only included studies that overlapped a maximum of two calendar years in the model. In all models, we included a random intercept to account for variability between study sites, and a random slope to account for temporal variability within sites (using the temporal mid-point of each study [102]). 

#### 2.4.1. Data Exploration

We first investigated collinearity between the environmental variables computed for each risk factor using a Pearson correlation matrix. Almost all variables computed for a potential risk factor were highly collinear (Pearson’s R > 0.80). Therefore, to determine which variables to retain in the subsequent analyses for each risk factor, we fitted separate models and computed the associated odds ratio, Akaike Information Criterion (AIC) and weights (see Supplementary Material 2). Within each group, we identified the variable with the highest predictive potential (lowest AIC, highest AIC weight, and odds ratio confidence intervals excluding one) [103,104,105]. This method eliminates potential risk factors with little explanatory power, with the aim to fit models as parsimoniously as possible. At the end of the procedure, the median nitrate concentration, maximum silicate concentration, and number of days with a temperature above 30 °C were retained. The variables computed for the other risk factors (seabed depth, ultraviolet exposure, phosphate concentration) had non-significant odds ratios, and were excluded from the subsequent analyses (see Supplementary Material 2 for full details).

#### 2.4.2. Quantification of the Effect of Each Risk Factor

Using the selected environmental variables, we implemented a series of mixed-effect models to quantify the effects of various potential risk factors on the prevalence of fibropapillomatosis. To obtain an optimal model, we tested different combinations of the following risk factors: maturity of turtle (i.e., the study included immature, adult, or a mix of both), sea turtle status (live, stranded, or a mix of both), median nitrate concentration, maximum silicate concentration, number of days SST > 30 °C, presence/absence of wrasse and leeches, and the mid-year point of each study. The body size of sea turtles has a well-documented positive effect on the prevalence of fibropapillomatosis [26,106]; thus, this variable was included in all models (i.e., it was used as a null model to compare the effect of other risk factors). These models were fitted using maximum likelihood (ML). The model with the lowest AIC and highest AIC Weight was retained. 

The optimal model was then refitted using the restricted maximum likelihood method (REML) to generate predictions and interpret its coefficients [102]. To visualize the effect of a risk factor on fibropapillomatosis prevalence, we computed marginal effects, which involve varying one risk factor at a time, while keeping all other factors constant (see [107]). The computation of marginal effects allows potential trends in the dataset to be visually represented. Since the computation of p-values is not reliable for mixed-effect models (see [108,109]), we reported the 95% confidence intervals (95% CI) of odds ratios (OR). Odds ratios were considered significant if one 95% CI did not overlap with another [104]. Means were reported with their standard deviation and associated range. All models were fitted in R software V.4.0.2 [110] using the glmmTMB package [111], the marginal effect was fitted using the ggeffects package [107] and usual validation diagnostics for mixed-effect models (see [102]) were performed using the DHARma package [112].

## 3. Results

**Figure 1:**
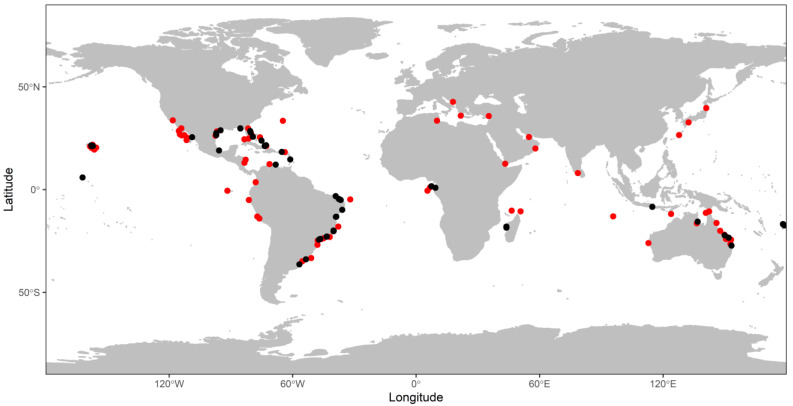
Location of the study sites (black circles) included in our meta-analysis of green turtles (*Chelonia mydas*) frequenting foraging areas for which the prevalence of fibropapillomatosis was reported. Red circles represent green turtle foraging sites adapted from the most recent comprehensive global meta-analysis of diet for this species [113], showing the spread of our sampling effort.

We retained 31 published studies in our meta-analysis, identifying 49 study sites (Figure 1) and 265 prevalence measurements (computed from a mean sample size of 187 ± 387 individuals, range:11–4407, for a total of 49,606 surveyed turtles globally) between 1994 and 2019. Fibropapillomatosis prevalence was estimated once at 23 sites, and between 2 and 21 times (average 7.6 ± 4.3) at the other 26 sites (see Supplementary Material 1 for a list of study sites.). Out of the 265 prevalence measurements, 38 (14%) were calculated on adults, 71 (27%) on immature turtles, and 156 (59%) on a mix of adult and immature turtles. Similarly, 225 (85%) prevalence measurements were calculated on live turtles, 13 (5%) on stranded turtles, and 27 (10%) on a mix of live and stranded turtles. 

**Figure 2:**
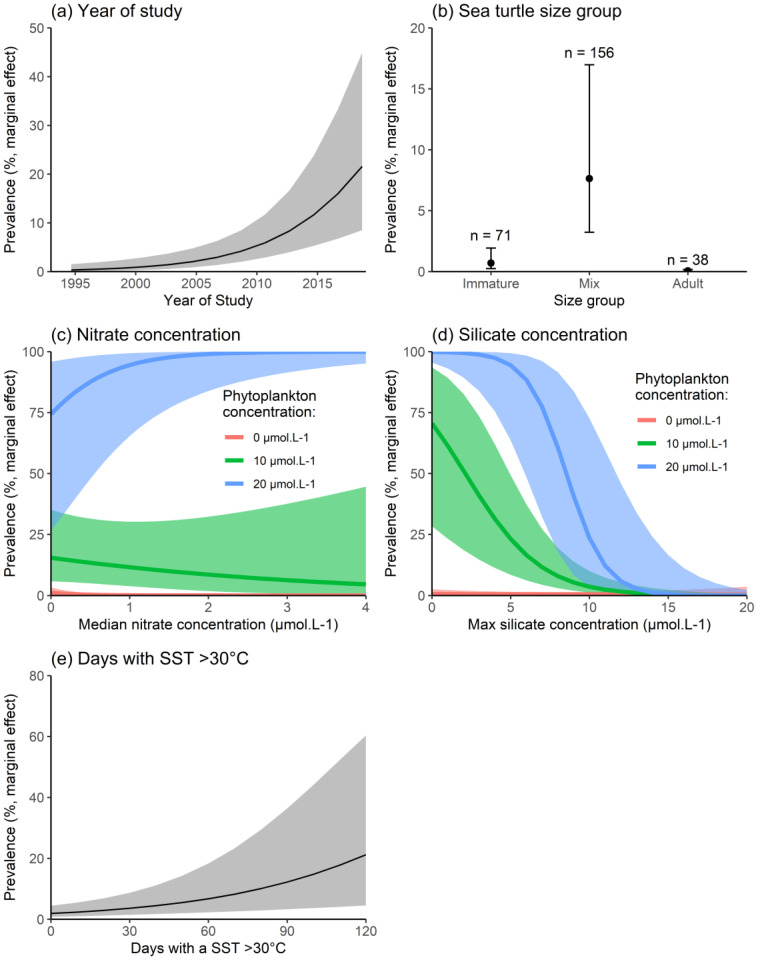
Marginal effect of fibropapillomatosis risk factors on green turtles (*Chelonia mydas*) computed on a global scale from the optimal logistic regression mixed-effect model for: (**a**) mid-year of each survey; (**b**) turtle size group; (**c**) Median nitrate concentration in seawater; (**d**) Maximum silicate concentration in sea water; and (**e**) number of days with a sea surface temperature >30 °C. Marginal effects indicate by how much, on average, the prevalence of fibropapillomatosis is expected to increase when exposure to a given risk factor increases (while keeping all other risk factors constant). In (**c**,**d**), a high concentration of nitrate and low concentration of silicate favour the proliferation of non-siliceous phytoplanktonic blooms (i.e., toxic dinoflagellates).

**Figure 3:**
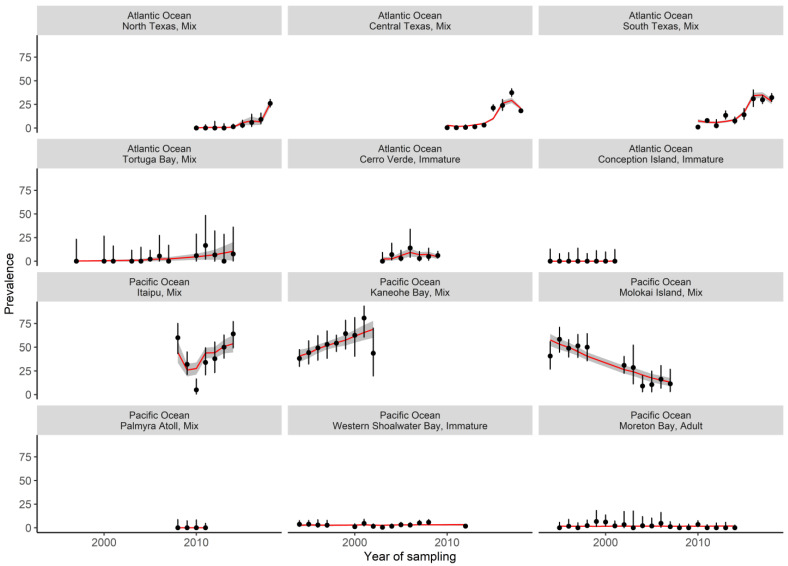
Measured fibropapillomatosis prevalence (black dots) and associated 95% CI intervals (black error bars) for 12 foraging sites of green turtles (*Chelonia mydas*) globally. The prevalence estimated by the optimal mixed-effect model is represented by a red line, and the associated 95% CI as grey shading. The size group (immature, adult, mix) of surveyed green turtles is stated with the site name. Sources for prevalence data: north, south and central Texas, USA [71]; Palmyra Atoll, USA [58]; Cerro Verde, Uruguay [72]; Conception Island, Bahamas [73]; Moreton Bay and Western Shoalwater Bay, Australia [76], Kaneohe Bay and Molokai Island, USA [52]; Tortuga Bay, Puerto Rico [60]; and Itapu, Brazil [51].

On a global scale, the prevalence of fibropapillomatosis increased in green turtles, on average, between 1994 and 2019 (OR: 1.20, 95% CI: 1.10–1.32, Figure 2a). As expected, there were significant differences in prevalence between the body size groups. Studies including a mix of adult and immature turtles reported the highest prevalence, on average. Studies that only evaluated immature (OR: 0.08, 95% CI: 0.01–0.21) or adult turtles (OR: 0.01, 95% CI: 0.00–0.02) had a lower prevalence compared to studies that included both (Figure 2b). There was a strong interaction between nutrient availability, phytoplankton concentration, and fibropapillomatosis prevalence (OR:0.96, 95% CI: 0.94–0.99 for the interaction between maximum silicates and phytoplankton concentrations; OR:1.24, 95% CI: 1.14–1.34 for the interaction between median nitrates and phytoplankton concentrations. Supplementary Tables S9 and S10). Prevalence was higher in areas with combined low silicate concentrations, high nitrate concentrations, and high phytoplankton concentrations. Regardless of nitrate and silicate concentrations, low fibropapillomatosis prevalence was observed in areas of low phytoplankton concentrations (Figure 2c,d). We also found moderate evidence that prevalence increases when the number of days of SST > 30 °C rises (OR:1.02, 95% CI: 1.01–1.04, Figure 2e, Supplementary Tables S9 and S10). Overall, there was good agreement between observed and modelled prevalence globally (Figure 3).

There was no evidence that fibropapillomatosis prevalence in green turtles at a global scale was impacted by: exposure to ultraviolet radiation (OR: 1.00, 95% CI: 1.00–1.00 for mean annual UV exposure), seabed depth (OR: 0.99, 95% CI: 0.84–1.17 for mean depth), phosphate concentration (OR: 0.44, 95% CI: 0.03–6.75 for mean phosphate concentration), or sea turtle status (see Supplementary Table S9). Similarly, we found no evidence that the presence of saddleback wrasses (OR: 6.22, 95% CI: 0.34–113.6) or leeches (OR: 0.49, 95% CI: 0.10–2.42) increased the prevalence of fibropapillomatosis on a global scale.

## 4. Discussion

This study delineates the cancer risk landscape of green turtles by combining a global meta-analysis with models of potential risk factors. Our results indicate that fibropapillomatosis is more prevalent in areas exposed to greater eutrophication, with cancer promotors (e.g. biotoxins) appearing to be present in these areas. In contrast, there was no evidence that wrasses and leeches, or UV exposure, contributed to the observed prevalence of fibropapillomatosis. Our findings highlight the risk factors that managers should monitor at all coastal foraging sites of green turtles globally [113] to mitigate fibropapillomatosis, and could potentially be used to forecast the likelihood of incidence.

### 4.1. Effect of Eutrophication

Our results indicate that high prevalence of fibropapillomatosis is associated with an exposure to toxic phytoplankton blooms. While the abundance of toxic phytoplankton could not be directly measured in our study, nutrient availability is usually a good indicator of the risk of toxic blooms [114]. Indeed, in marine ecosystems, high concentrations of nitrogen and phosphorus promote the development of diatom blooms until silicates are depleted [115,116]. Consequently, nonsiliceous taxa (such as cyanobacteria and dinoflagellates), which are more efficient at growing in nutrient-poor and silica-limited environments, tend to outcompete diatoms to become dominant, and form toxic blooms covering areas up to several hundreds of kilometers [117,118]. We found that the prevalence of fibropapillomatosis was greater in areas with both high phytoplankton concentration and low silicate concentration. In addition, extended periods of high temperatures increase stratification in the water column, potentially favoring toxic phytoplanktonic blooms [116]. Our results suggest that green turtles exposed to high oceanic temperatures (> 30 °C) were associated with a higher prevalence of fibropapillomatosis. This finding supports the observation that rehabilitating turtles (here sea turtles treated at the Georgia Sea Turtle Center, Jekyll Island, USA) tend to develop new tumors faster during warmer months [36]. 

Thus, fibropapillomatosis is likely associated with exposure to harmful algal blooms that are promoted by higher water temperatures and eutrophication. Indeed, biotoxin-producing algae can generate large quantities of contact irritants causing inflammatory responses (a cancer promoter) or neurotoxins [88,119]. Exposure to these biotoxins likely occurs when green turtles ingest seagrass, during gular pumping, or are possibly inhaled during pre-dive breathing [91,120,121]. Certain molecules, such as brevetoxin (a neurotoxin), accumulate in the plasma of green turtles, and might bind with albumin, causing long-term inflammation and exacerbating oxidative stress responses (another cancer promotor [91,120,122,123]) 

### 4.2. Transmission of the Virus

The most likely route of transmission for ChHV5 is through the shedding of viral particles in the water column [100,124]. At a local scale, the dispersal of such particles depends on certain key environmental factors, such as oceanic currents (which physically disperse the virus) and sea water temperatures (which affect infectivity and virulence). For example, lung–eye–trachea disease-associated herpesvirus remains infective in natural sea water for at least 120 h at 23 °C [125]. Similarly, the infectivity of another herpesvirus that causes grey-patch disease in green turtles increases as temperature rises from 25 °C to 30 °C [93], likely because higher temperatures enhance herpesviruses replication. If ChHV5 particles that shed in the water column have similar dispersal traits, infection would primarily occur at a given coastal foraging site, with relatively limited transfer across spatially distinct sites (as suggested by genetic analyses of the virus in infected sea turtles [77,100,126]). Alternatively, transfer across sites might be driven by potential mechanical vectors, like wrasses or leeches. However, we found no evidence, on a global scale, that either of these two vectors are associated with the prevalence of fibropapillomatosis. These potential mechanical vectors may contribute to the transmission of the virus at a local scale, because it has been detected in them [97,98,99]. Thus, continued monitoring of the relationship between temperature and fibropapillomatosis is required, as well as studies on the mechanisms driving the transmission routes and rates of this virus across foraging sites, including data on leech loads on turtles, the frequency of fish cleaning station visits, and the frequency of movement and interactions with turtles from other sites (which can be quantified, see [127,128,129]). Knowledge on the ChHV5 variants documented globally also remains limited, including transmission rates. Different strains could drive different immune responses in turtles across regions, as observed between the Pacific (Hawaii) and Atlantic (Florida) green turtle stocks, potentially driving differences in prevalence [30,130]. Thus, epidemiological studies on the co-evolution of the virus and its hosts are required across regions.

### 4.3. Implications for the Conservation of Green Turtles

Efficient treatments of fibropapillomatosis in green turtles are lacking [82]. While the regression of tumors has been detected in some individuals in the wild [37], many die, with the surgical removal of tumors having limited effect because they often regrow [131]. In particular, survival is strongly associated with the body parts on which tumors develop [132]. For example, sea turtles with ocular tumors are eight times more likely to die compared to those with tumors on other body parts [36]. Our study also showed that environmental factors exacerbate the progression and prevalence of disease; thus, actions towards restoring ecosystem quality (for example, by reducing eutrophication) could help to limit, or entirely prevent outbreaks of fibropapillomatosis in sea turtles. For instance, actively monitoring the cancer risk landscape in areas where green turtles forage could help detect emerging hotspots. Focus could be placed on mitigating nutrient input (and hence toxic phytoplanktonic blooms) to these areas by using indicators to predict and forestall fibropapillomatosis outbreaks, implementing dynamic ocean management to prevent the overlap of threatened marine wildlife, similar to that already implemented for fisheries and shipping routes [133,134].

### 4.4. Limitations and Conclusions

The presence of a significant temporal trend in the prevalence of fibropapillomatosis in our best model indicates that some risk factors have yet to be identified for fibropapillomatosis in green turtles. We found that the reported prevalence was higher in studies with a mix of immature and adult turtles, as previously documented [26,106]. However, data on separate immature and adult size classes were limited, with previous comparisons between studies using size as a continuous variable, rather than a categorical classification as done in this study, being difficult to conduct. The low prevalence of fibropapillomatosis in studies of immature turtles might be attributed to relatively smaller (i.e., younger) individuals being encountered, which are less likely to harbor tumors. In contrast, studies that evaluated both immature and mature sea turtles likely screened for a larger proportion of the population (and age classes), leading to more individuals with fibropapillomatosis being detected. Cross-discipline studies integrating information on epidemiology, environment, and turtle characteristics (e.g. demography, genetic diversity, behavior, movement and habitat use) at large scales are required to establish causality links [46,135,136,137]. There is currently no evidence that organic chemical contamination increases the prevalence of fibropapillomatosis in sea turtles [40,138]. However, data are lacking on chemical contamination on a global scale [139], particularly for emerging contaminants.

Our study focused on establishing trends in the prevalence of fibropapillomatosis for green turtles on a global scale, and could be replicated at different scales or on a different species to facilitate management actions (i.e., from site scale to regional management unit scale [RMU] [140]). Because fibropapillomatosis appears to be associated with multifactorial risks [39], with sea turtles inhabiting a complex and dynamic cancer risk landscape [23], it is important to assimilate longitudinal datasets investigating multiple risk factors as a whole (e.g., phytoplankton bloom occurrence, composition, and associated biotoxins), along with any other environmental variables of relevance at a given site (e.g., other cancer promotors in the environment, ChHV5 shedding rates from environmental DNA [100], genetics or immune responses of sea turtles, emerging contaminants). The sharing of holistic datasets collected from multiple study sites within a strong collaborative network could facilitate rapid advances in our understanding of the disease, similar to those developed for cancer in humans [141] and transmissible cancers in wildlife [46]. In conclusion, through identifying key environmental parameters associated with the prevalence of fibropapillomatosis, this study provides a basis for developing direct monitoring and management strategies to mitigate this cancer.

## Data Availability

All data sources used in this publication are cited in the manuscript or in the Supplementary Materials.

## Supplementary Materials

The following are available online at https://www.mdpi.com/article/10.3390/pathogens10101295/s1, Supplementary Materials 1 “List of publication included in the meta-analysis”, Supplementary Materials 2 “Selection of variables for inclusion in the mixed effect models”, Supplementary Result 1 “Summary the logistic mixed effect models”. Supplementary Table S1: Summary of the published literature (1994–2019) on fibropapillomatosis in green sea turtles at foraging areas globally and included in the meta-analysis; Supplementary Table S2: Summary results of the models used to explore the variables derived from nitrate concentration data; Supplementary Table S3: Summary results of the models used to explore the variables derived from phosphate concentration data; Supplementary Table S4: Summary results of the models used to explore the variables derived from silicate concentration data; Supplementary Table S5: Summary results of the models used to explore the variables derived from phytoplanktonic concentration data; Supplementary Table S6: Summary results of the models used to explore the variables derived from seabed depth data; Supplementary Table S7: Summary results of the models used to explore the variables derived from ultraviolet exposure data; Supplementary Table S8: Summary results of the models used to explore the variables derived from SST data; Supplementary Table S9: Summary table of the logistic mixed effect models used to investigate the effect of risk factors on fibropapillomatosis in green sea turtles to determine an optimal model; Supplementary Table S10: Odd ratios computed from the optimal model linking fibropapillomatosis prevalence in green sea turtles to a range of risk factors.
