# Supplementary material for: Sea Turtles in the Cancer Risk Landscape: A Global Meta-Analysis of Fibropapillomatosis Prevalence and Associated Risk Factors
Source: Pathogens. 2021 Oct 8;10(10):1295. doi: 10.3390/pathogens10101295 (PMC8540842; doi:10.3390/pathogens10101295)
Supplement: Supplementary file 1 [file pathogens-10-01295-s001.zip › pathogens-1357248-SI.pdf]

Sea turtles in the cancer risk landscape: a global meta-analysis of fibropapillomatosis prevalence and associated risk factors

Running title: Cancer risk factors in green sea turtles

Antoine M. Dujon[1,2,3*], Gail Schofield[4], Roberto Venegas[1], Frédéric Thomas[2,3], Beata Ujvari[1,2]

[1]Deakin University, Geelong, School of Life and Environmental Sciences, Centre for Integrative Ecology, Waurn Ponds, Vic 3216, Australia

[2]CANECEV-Centre de Recherches Ecologiques et Evolutives sur le cancer (CREEC), Montpellier 34090, France.

[3]CREEC, UMR IRD 224-CNRS 5290-Université de Montpellier, Montpellier, France

[4]School of Biological and Chemical Sciences, Queen Mary University of London, London E1 4NS, UK

Supplementary Material 1: List of publication included in the meta-analysis.

Supplementary Table S1: Summary of the published literature (1994-2019) on fibropapillomatosis in green sea turtles at foraging areas globally. Information on associated prevalence, sample size and sampling period is listed, and was in included in the meta-analysis. The maturity of sea turtles was determined based on the information and classification provided by the authors of each publication for each study site.

Study Site	Sample Size	Prevalence (%)	Time series length	Size group	Reference
Bahia, Brazil	1255	24	2009-2010	Immature	[1]
Bali, Indonesia	4407	21.5	1994	Mix	[2]
Bimini, Bahamas	58	24.1	2016-2016	Immature	[3]
Brazil Stretch A	284±48 (236-339)	24.6±9.8 (15.7-38.6)	2012-2015	Mix	[4]
Brazil Stretch B	176±27 (154-216)	10.3±6.5 (3.7-17.2)	2012-2015	Mix	[4]
Brazil Stretch C	68±21 (52-98)	8.1±5.5 (0-11.6)	2012-2015	Mix	[4]
Ceara, Brazil	701	31.4	2009-2010	Immature	[1]
Central Texas	758±782 (165-2702)	11.9±13.7 (0.4-37.4)	2010-2018	Mix	[5]
Cerro Verde	72±62 (27-209)	5.4±4.4 (0-14)	2003-2009	Immature	[6]
Conception Island	49±21 (25-100)	0	1994-2001	Immature	[7]
Corisco Bay	69±38 (33-151)	18.1±5.6 (9.7-27.3)	1998-2006	Mix	[8]
Espirito Santo, Brazil	163	58.3	2007-2008	Mix	[9]
Espirito Santo, Brazil	2261	12.7	2009-2010	Immature	[1]
Heron Island	142±27 (116-173)	0.5±0.4 (0-0.8)	1994-1999	Adult	[10]
Heron Island	269±43 (230-331)	0.4±0.3 (0-0.8)	1994-1999	Immature	[10]
Itaipu	50±13 (39-71)	40.4±20 (5-64)	2008-2014	Mix	[11]
Kaneohe Bay	62±45 (16-142)	54.5±13.1 (38.3-80.8)	1994-2002	Mix	[12]
La Martinique	150	16.7	2019	Mix	[13]
Bonaire	114±53 (81-259)	14.1±13.3 (0-33.7)	2006-2014	Mix	[14,15]
Makogai Island	46	0	2005	Immature	[16]
Mar de Ajó	27	0	2004-2005	Mix	[17]
Molokai Island	60±32 (21-125)	32.4±18.5 (9.1-58.3)	1994-2007	Mix	[12]
Moreton Bay	76±36 (19-172)	1.8±2.1 (0-6.7)	1995-2014	Adult	[10]
Moreton Bay	250±90 (67-480)	16.3±4.9 (4-21.8)	1994-2014	Immature	[10]
N1 Region Brazil	49±24 (26-79)	8.5±1.8 (6.7-11.2)	1994-1998	Mix	[9]
N2 Region Brazil	25±6 (19-34)	5.2±6.1 (0-11.7)	1994-1998	Mix	[9]
Navachiste Lagoon	24	0	2005-2005	Immature	[18]
Nearshore Reef, Florida	256	14.8	1998-1999	Immature	[19]

Niteroi, Southeast Brazil	131	31.3	2008-2010	Immature	[11]
North Texas, Mix	156±136 (51-494)	5.1±8.5 (0-26.1)	2010-2018	Mix	[5]
Northestern Gulf of Mexico	434	6	2010	Mix	[20]
Nosy Andrano Island	12	0	2012	Mix	[21]
Nosy Marify Island	105	1.9	2012	Mix	[21]
Nosy Maroantaly Island	159	10.7	2012	Mix	[21]
Pala‘au, Hawaii	108	50	1997-1998	Mix	[22]
Palmyra Atoll	53±17 (41-78)	0	2008-2011	Mix	[23]
Príncipe Island	50	32	2009	Mix	[24]
Puerto Manglar	26±10 (11-41)	15.8±22.5 (0-79.2)	1997-2014	Mix	[25]
Sao Paulo, Brazil	1971	4.6	2009-2010	Immature	[9]
Sir Edward Pellew Islands	102	0	2002	Mix	[26]
South Texas	348±283 (79-996)	15.5±12.4 (1-32.2)	2010-2018	Mix	[5]
St. Joseph Bay	387	0	2000	Mix	[27]
State of Bahia	52±19 (31-82)	4.1±5.1 (0-12.4)	1994-1999	Mix	[9]
State of Espírito Santo	42±21 (11-72)	9.8±7.5 (2-21.2)	1994-1999	Mix	[9]
State of São Paulo	389±187 (229-656)	4.1±4 (1.2-10.4)	1994-1999	Mix	[9]
Tortuga Bay	19±10 (11-46)	3.4±5 (0-16.7)	1997-2014	Mix	[25]
Trident Submarine Basin	82	0	1998-2000	Immature	[19]
Turk and Caicos islands	239	13.4	2008-2010	Mix	[28]
Veracruz Reef National Park	41	12.2	2017-2018	Mix	[6]
Western Shoalwater Bay	175±37 (120-234)	0.2±0.3 (0-0.7)	1994-2012	Adult	[10]
Western Shoalwater Bay	253±107 (101-453)	2.9±1.5 (0.5-5.8)	1994-2012	Immature	[10]
Yadua Island	64	0	2005	Immature	[16]

References

1. Rodenbusch, C.R.; Baptistotte, C.; Werneck, M.R.; Pires, T.T.; Melo, M.T.D.; De Ataíde, M.W.; Dos Reis, K.D.H.L.; Testa, P.; Alieve, M.M.; Canal, C.W. Fibropapillomatosis in green turtles *Chelonia mydas* in Brazil: Characteristics of tumors and virus. *Dis. Aquat. Organ.* **2014**, *111*, 207–217, doi:10.3354/dao02782.

2. Adnyana, W.; Ladds, P.W.; Blair, D. Observations of fibropapillomatosis in green turtles (*Chelonia mydas*) in Indonesia. *Aust. Vet. J.* **1997**, *75*, 736–742, doi:10.1111/j.1751-0813.1997.tb12258.x.

3. Gillis, J.A. Foraging Ecology and Diet Selection of Juvenile Green Turtles (*Chelonia mydas*) in the Western Bahamas: Insights from Stable Isotope Analysis and Prey Mapping, Master Thesis, Florida State University, 2018.

4. Silva-Júnior, E.S. Da; De Farias, D.S.D.; Costa Bomfim, A. Da; Boaviagem Freire, A.C. Da; Revorêdo, R.Â.; Rossi, S.; Matushima, E.R.; Hildebrand Grisi-Filho, J.H.; De Lima Silva, F.J.; Gavilan, S.A. Stranded Marine Turtles in Northeastern Brazil: Incidence and Spatial-Temporal Distribution of Fibropapillomatosis. *Chelonian Conserv. Biol.* **2019**, *18*, 249–258, doi:10.2744/CCB-1359.1.

5. Shaver, D.J.; Walker, J.S.; Backof, T.F. Fibropapillomatosis prevalence and distribution in green turtles *Chelonia mydas* in Texas (USA). *Dis. Aquat. Organ.* **2019**, *136*, 175–182, doi:10.3354/dao03403.

6. López-Mendilaharsu, M.; Vélez-Rubio, G.M.; Lezama, C.; Aisenberg, A.; Bauzá, A.; Berrondo, L.; Calvo, V.; Caraccio, N.; Estrades, A.; Hernández, M.; et al. Demographic and tumour prevalence data for juvenile green turtles at the Coastal-Marine Protected Area of Cerro Verde, Uruguay. *Mar. Biol. Res.* **2016**, *12*, 541–550, doi:10.1080/17451000.2016.1169294.

7. Bjorndal, K.A.; Bolten, A.B.; Chaloupka, M.Y. Evaluating trends in abundance of immature green turtles, *Chelonia mydas*, in the Greater Caribbean. *Ecol. Appl.* **2005**, *15*, 304–314, doi:10.1890/04-0059.

8. Formia, A.; Deem, S.; Billes, A.; Ngouessono, S.; Parnell, R.; Collinis, T.; Sounguet, G.P.; Gibudi, A.; Villarubia, A.; Balazs, G.H.; et al. Fibropapillomatosis confirmed in *Chelonia mydas* in the Gulf of Guinea, West Africa. *Mar. Turt. Newsl.* **2007**, *116*, 20–22.

9. Baptistotte, C.; Sclfoni, J.T.; Gallo, B.M.G.; dos Santos, A.S.; de Castilhos, J.C.;

Lima, E.H.S.M.; Bellini, C.; Barata, P.C.R. Prevalence of sea turtle fribropapillomatosis in Brazil. In Proceedings of the 21st Annual Symposium on Sea Turtle Biology and Conservation; Philadelphoa, Pensilvania, USA, 2001.

10. Jones, K. Environmental influences on the epidemiology of fibropapillomatosis in green turtles (*Chelonia mydas*) and consequences for management of inshore areas of the Great Barrier Reef., James Cook University, 2019.

11. Tagliolatto, A.B.; Guimarães, S.M.; Lobo-Hajdu, G.; Monteiro-Neto, C. Characterization of fibropapillomatosis in green turtles *Chelonia mydas* (Cheloniidae) captured in a foraging area in southeastern Brazil. *Dis. Aquat. Organ.* **2016**, *121*, 233–240, doi:10.3354/dao03020.

12. Murakawa, S.K.K. Hawaiian Archipelago Fibropapillomatosis Data. In Proceedings of the Proceedings of the 2015 International Summit on Fibropapillomatosis: Global Status, Trends, and Population Impacts; Honolulu, Hawaii, USA, 2015; pp. 36–56.

13. Liebart, M. Photo-identification des tortues vertes (*Chelonia mydas*) et son application dans l’indice d’abondance ou de fidélité aux sites d’alimentation en Martinique, 2019.

14. Patricio, A.R.; Centre, E.S. Fibropapillomatosis in Marine Turtles of the Caribbean Region : the Case Study of Puerto Rico. In Proceedings of the International Summit on Fibropapillomatosis: Global Status, Trends, and Population Impacts; Honolulu, Hawaii, 2016.

15. Mil van, C. *Fibropapillomatosis affecting green turtles (*Chelonia mydas*).*; Sea Turtle Conservation Bonaire Research Report, 2014;

16. Piovano, S.; Lemons, G.E.; Ciriyawa, A.; Ciriyawa, A.; Batibasaga, A.; Seminoff, J.A. Diet and recruitment of green turtles in Fiji, South Pacific, inferred from in-water

capture and stable isotope analysis. *Mar. Ecol. Prog. Ser.* **2020**, *640*, 201–213, doi:10.3354/meps13287.

17. Albareda, D.A.; Garne, M.; Prosdocimi, L.; Rodriguez, H.S.; Di, P.J.L.; Loureiro, J. Pathological studies in green sea turtles (*Chelonia mydas*) and loggerhead sea turtles (*Caretta caretta*) from the northern coastal area of Buenos Aires, Argentina. In Proceedings of the Proceedings of the Twenty-Seventh Annual Symposium on Sea Turtle Biology and Conservation; Myrtle Beach, South Carolina, USA, 2008.

18. Mejía-Radillo, R.Y.; Zavala-Norzagaray, A.A.; Alicia Chávez-Medina, J.; Alonso Aguirre, A.; Escobedo-Bonilla, C.M. Presence of chelonid herpesvirus 5 (ChHV5) in sea turtles in northern Sinaloa, Mexico. *Dis. Aquat. Organ.* **2019**, *132*, 99–108, doi:10.3354/dao03313.

19. Hirama, S.; Ehrhart, L.M. Description, prevalence and severity of green turtle fibropapillomatosis in three developmental habitats on the East Coast of Florida. **2007**, *70*, 435–448.

20. Avens, L.; Goshe, L.R.; Harms, C.A.; Anderson, E.T.; Hall, A.G.; Cluse, W.M.; Godfrey, M.H.; McNeill, J.B.; Stacy, B.; Bailey, R.; et al. Population characteristics, age structure, and growth dynamics of neritic juvenile green turtles in the northeastern Gulf of Mexico. *Mar. Ecol. Prog. Ser.* **2012**, *458*, 213–229, doi:10.3354/meps09720.

21. Campillo, A. *Projet « Origine , Répartition et Evolution du Fibropapillomas aux îles Barren »*; Association Caouanne Research Report, 2012;

22. Work, T.M.; Balazs, G.H. Relating tumor score to hematology in green turtles with fibropapillomatosis in Hawaii. *J. Wildl. Dis.* **1999**, *35*, 804–807, doi:10.7589/0090-3558-35.4.804.

23. Sterling, E.J.; McFadden, K.W.; Holmes, K.E.; Vintinner, E.C.; Arengo, F.; Naro-Maciel, E. Ecology and conservation of marine turtles in a central pacific foraging ground. *Chelonian Conserv. Biol.* **2013**, *12*, 2–16, doi:10.2744/CCB-1014.1.

24. Loureiro, N. de S.; Damião Matos Presence of fibropapillomatosis in green turtles *Chelonia mydas* at Príncipe Island in the Gulf of Guinea. *Arquipélago. Life Mar. Sci.* **2009**, *26*, 79–83.

25. Patrício, A.R.; Diez, C.E.; Van Dam, R.P.; Godley, B.J. Novel insights into the dynamics of green turtle fibropapillomatosis. *Mar. Ecol. Prog. Ser.* **2016**, *547*, 247–255, doi:10.3354/meps11644.

26. Hamann, M.; Schäuble, C.; Simon, T.; Evans, S. Demographic and health parameters of green sea turtles *Chelonia mydas* foraging in the Gulf of Carpentaria, Australia. *Endanger. Species Res.* **2006**, *2*, 81–88, doi:10.3354/esr002081.

27. Foley, A.M.; Singel, K.E.; Dutton, P.H.; Summers, T.M.; Redlow, A.E.; Lessman, J. Characteristics of a green turtle (*Chelonia mydas*) assemblage in northwestern Florida determined during a hypothermic stunning event. *Gulf Mex. Sci.* **2007**, *25*, 131–143, doi:10.18785/goms.2502.04.

28. Stringell, T.B.; Clerveaux, W. V.; Godley, B.J.; Phillips, Q.; Ranger, S.; Richardson, P.B.; Sanghera, A.; Broderick, A.C. Fisher choice may increase prevalence of green turtle fibropapillomatosis disease. *Front. Mar. Sci.* **2015**, *2*, 1–8, doi:10.3389/fmars.2015.00057.

Supplementary Material 2: Selection of variables for inclusion in the mixed effect models

To explore which environmental variables computed for each risk factor should be retained to explore their effect on fibropapillomatosis prevalence in green sea turtles, we used a series of logistic mixed effect regression models (using the logit link function). Within each group of variables, the one with the highest explanatory power was retained (lowest AIC value and highest AIC Weight). In addition, a variable was only retained if the 95% confidence limit of its odds ratios did not include one.

Nitrates (NO_3^-):

Median NO_3^- concentration (in µmol.L^{-1}) was retained as a variable for inclusion in the model (Supplementary Table S1).

Supplementary Table S2: Summary results of the models used to explore the variables derived from nitrate concentration data. Significant odd ratios are presented in bold.

Variable	df	AIC	AIC Weights	Odds Ratio (95% CI)
Minimum NO_3^-	5	2511.2	0	**0.13 (0.03-0.64)**
Maximum NO_3^-	5	2503	0	**1.38 (1.19-1.58)**
Mean NO_3^-	5	2409.3	0	**0.06 (0.04-0.11)**
Median NO_3^-	5	2389.4	1	**0.11 (0.08-0.17)**
Standard Deviation NO_3^-	5	2505.2	0	**0.07 (0.010-0.30)**

Phosphates (PO_4^-):

There were no significant odds ratios computed using the variables derived from the phosphate concentration data. Phosphate was therefore excluded from the mixed effect models investigating the risk factors of fibropapillomatosis (Supplementary Table S3).

Supplementary Table S3: Summary results of the models used to explore the variables derived from phosphate concentration data. No significant odds ratios were detected.

Variable	df	AIC	AIC Weights	Odds Ratio (95% CI)
Minimum PO_4^-	5	2516.6	0.05	1.51 (0.08-30.27)
Maximum PO_4^-	5	2517	0.04	0.58 (0.08-4.26)
Mean PO_4^-	5	2516.7	0.05	0.73 (0.05-11.36)
Median PO_4^-	5	2516.5	0.05	0.55 (0.04-8.33)
Standard Deviation PO_4^-	5	2511	0.81	8623486.27 (0-2755140187615426560)

Silicates (Si):

Maximum silicate concentration (in µmol.L^{-1}) was retained as a variable for inclusion in the model (Supplementary Table S4).

Supplementary Table S4: Summary results of the models used to explore the variables derived from silicate concentration data.

Variable	df	AIC	AIC Weights	Odds Ratio (95% CI)
Minimum Si	5	2521.3	0	1.07 (0.84-1.38)
Maximum Si	5	2481.6	1	0.73 (0.66-0.82)
Mean Si	5	2506.9	0	0.73 (0.61-0.87)
Median Si	5	2514.1	0	0.81 (0.70-0.94)
Standard Deviation Si	5	2519	0	1.14 (0.48-2.75)

Phytoplankton concentration

The maximum phytoplankton (PHY) concentration (in µmol.L^{-1}) was retained as a variable for inclusion in the model (Supplementary Table 5).

Supplementary Table S5: Summary results of the models used to explore the variables derived from phytoplanktonic concentration data. Significant odd ratios are presented in bold.

Variable	df	AIC	AIC Weights	Odds Ratio (95% CI)
Minimum PHY	5	2508.1	0.16	**7.1 (2.12-23.81)**
Maximum PHY	5	2504.8	0.83	**1.28 (1.14-1.46)**
Mean PHY	5	2513.7	0.01	**1.62 (1.14-2.32)**
Median PHY	5	2520.2	0	1.17 (0.84-1.62)
Standard Deviation PHY	5	2518.2	0	1.07 (0.28-4.1)

Seabed Depth:

There were no significant odds ratios computed using the metrics derived from seabed depth data (Supplementary Table S6). Seabed depth was therefore excluded from the mixed effect models investigating the risk factors of fibropapillomatosis.

Supplementary Table S6: Summary results of the models used to explore the variables derived from seabed depth data. No significant odds ratios were detected.

Variable	df	AIC	AIC Weights	Odds Ratio (95% CI)
Mean Depth	5	2522.3	0.61	0.99 (0.84-1.17)
Median Depth	5	2523.2	0.39	1.00 (0.89-1.12)

Ultraviolet exposure:

There were no significant odds ratios computed using the variables derived from ultraviolet exposure data (Supplementary Table S7). Seabed depth was therefore excluded from the mixed effect models investigating the risk factors of fibropapillomatosis.

Supplementary Table S7: Summary results of the models used to explore the variables derived from ultraviolet exposure data. No significant odds ratios were detected.

Variable	df	AIC	AIC Weights	Odds Ratio (95% CI)
Min Exposure	5	2532.9	0	1.00 (1.00-1.00)
Max Exposure	5	2530.2	0	1.00 (1.00-1.00)
Mean Exposure	5	2532	0	1.00 (1.00-1.00)
Seasonality	5	2519.4	0.99	0.85 (0.43-1.7)

Sea surface temperature:

The number of days with an SST >30°C was retained as a variable for inclusion in the model (Supplementary Table 8).

Supplementary Table S8: Summary results of the models used to explore the variables derived from SST data. Significant odd ratios are presented in bold.

Variable	df	AIC	AIC.Weights	Odds Ratio (95%CI)
Min Temperature	5	2518.3	0	**1.08 (1.02-1.15)**
Max Temperature	5	2513.3	0	**1.22 (1.07-1.38)**
Mean Temperature	5	2509.3	0	**1.30 (1.13-1.49)**
Median Temperature	5	2511.7	0	**1.20 (1.07-1.32)**
Standard Deviation Temperature	5	2487.4	0	**0.03 (0.01-0.11)**
N Days >20°C	5	2528	0	1.00 (1.00 -1.01)
N Days >21°C	5	2530	0	1.00 (1.00 -1.00)
N Days >22°C	5	2522.1	0	1.01 (1.00-1.01)
N Days >23°C	5	2510.6	0	1.01 (1.00-1.01)
N Days >24°C	5	2525.6	0	1.00 (1.00 -1.01)
N Days >25°C	5	2528.4	0	1.00 (1.00-1.00)
N Days >26°C	5	2526.5	0	1.00 (1.00-1.00)
N Days >27°C	5	2520.2	0	1.01 (1.00-1.01)
N Days >28°C	5	2514.7	0	1.01 (1.00-1.01)
N Days >29°C	5	2491	0	**1.01 (1.01-1.02)**
N Days >30°C	5	2459.5	1	**1.03 (1.03-1.04)**

Supplementary Results 1:

Supplementary Table S9: Summary table of the logistic mixed effect models used to investigate the effect of risk factors on fibropapillomatosis in green sea turtles to determine an optimal model. The model with the lowest AIC and the highest AIC Weight was retained. Maximum silicate concentration (Si), median nitrate concentration (NO3), number of days with an SST >30 °C (SST), maximum phytoplanktonic concentration (PHY). "*" denotes an interaction between the terms of the model.

**Variables**	**df**	**AIC**	**AIC Weights**
Size+PHY*Si+PHY*NO3+SST+Year	13	1901.3	0.96
Size+Si+PHY*NO3+SST+Year	12	1908.1	0.03
Size+PHY*Si+PHY*NO3+PHY*SST+Year	12	1910	0.01
Size+Si+NO3+SST+Year+PHY	11	1923.6	0
Size+Si*PHY+NO3+SST+Year	12	1925.4	0
Size+Condition+Leeches+Wrasses+Si+NO3+SST+Year	15	1930.4	0
Size+Si+NO3+SST+Year	10	1960.4	0
Size+Si+SST+Year	9	1971.4	0
Size+Condition+Si+SST+Year	11	1973.2	0
Size+NO3+SST+Year	9	1979.5	0
Size+Condition+NO3+Year	10	1980.2	0
Size+Condition+NO3+SST+Year	11	1982.1	0
Size+Condition+SST+Year	10	2039.9	0
Size+Condition+Si+Year	10	2057.8	0
Size+Condition+PHY+Year	10	2085.4	0
Size+Year+PHY	8	2085.6	0
Size+PHY	7	2097.8	0
Size+Condition+Year	9	2104.1	0
Size+Condition	8	2114.4	0
Size	6	2117.3	0
Size+Wrasses	7	2117.8	0
Size+Leeches	7	2118.5	0

Supplementary Table S10: Odd ratios computed from the optimal model linking fibropapillomatosis prevalence in green sea turtles to a range of risk factors. Significant odds ratios are in bold.

**Variable included in the model**	**Odds ratio (95%CI)**
Immature green turtles	(reference group for size class)
Mix of immature and adult green turtles	**11.78 (5.19-26.75)**
Adult green turtles	**0.09 (0.06-0.12)**
Year of sampling	**1.20 (1.10-1.32)**
Maximum silicates concentration (µmol.L^{-1})	0.97 (0.83-1.14)
Phytoplankton concentration (µmol.L^{-1})	**1.69 (1.37-2.09)**
Median nitrates concentration (µmol.L^{-1})	**0.09 (0.04-0.20)**
Number of days >30°C	**1.02 (1.01-1.04)**
Maximum silicates / phytoplankton concentrations interaction	**0.96 (0.94-0.99)**
Median nitrates / phytoplankton concentrations interaction	**1.24 (1.14-1.34)**
